# The correlation between sexual dysfunction and intimate partner violence in young women during pregnancy

**DOI:** 10.1186/s12914-020-00245-9

**Published:** 2020-09-14

**Authors:** Ellahe Bahrami_Vazir, Sakineh Mohammad-Alizadeh-Charandabi, Mahin Kamalifard, Fatemeh Ghelichkhani, Azam Mohammadi, Mojgan Mirghafourvand

**Affiliations:** 1 Department of midwifery, Faculty of Nursing and Midwifery, Ilam University of Medical Sciences, Ilam, Islamic Republic of Iran; 2 Department of Midwifery, Social Determinants of Health Research Centre, Faculty of Nursing and Midwifery, Tabriz University of Medical Sciences, Tabriz, Islamic Republic of Iran; 3 Department of Midwifery, Faculty of Nursing and Midwifery, Tabriz University of Medical Sciences, Tabriz, Islamic Republic of Iran; 4 Department of Midwifery, Imam Sajjad Hospital, Shahriar, Iran University of Medical Sciences, Tehran, Islamic Republic of Iran; 5 Department of Midwifery and Reproductive Health, Faculty of Nursing and Midwifery, Tehran University of Medical Sciences, Tehran, Islamic Republic of Iran

**Keywords:** Sexual dysfunction, Intimate partner violence, Pregnancy, Young, Women

## Abstract

**Background:**

Sexual function is one of the main aspects of life. Pregnancy affects sexual function. The aim of this study was to determine the sexual dysfunction in young pregnant women and its correlation with intimate partner violence (IPV).

**Methods:**

This cross-sectional study was conducted using two-stage cluster sampling method. The data were collected using a socio-demographic questionnaire, Female Sexual Function Index (FSFI), and Conflict Tactics Scale (CTS2). Multivariate logistic regression was used to determine the relationships between sexual dysfunction with IPV and socio-demographic factors.

**Results:**

The results of this study on 346 pregnant women aged 18–29 years showed that mean (SD = standard deviation) of the total sexual function score was 25.4 (5.9), within a possible score range of 2 to 36. About 66% of the young pregnant women had a sexual dysfunction. The lowest and the highest prevalence of sexual dysfunction were in sub domains of sexual satisfaction and sexual desire, respectively. The prevalence of overall IPV against young women was 63%. The most common type of IPV experienced by women was psychological aggression (56.6%). There were statistically significant relationships between sexual dysfunction and IPV (OR (Odds Ratio) = 0.4, 95% Confidence Interval = 0.2 to 0.6, *p* <  0.001), sufficiency of income for expenses (0.2, 0.1 to 0.6, *p* = 0.005), husband educational level (0.5, 0.3 to 0.9, *p* = 0.028) and marriage duration (1.9, 1.0 to 3.7, *p* = 0.044).

**Conclusions:**

Sexual dysfunction has a high prevalence in young pregnant women and IPV had correlation with sexual dysfunction. The routine screening for sexual dysfunction and IPV is recommended during pregnancy for detection and consulting.

## Background

Sexual health is a state of physical, emotional, mental and social well-being in relation to sexual function. Sexual function is a multidimensional phenomenon, which is affected by mental, social, economic, political, and cultural factors [1] as well as pregnancy [2]. Sexual function has changes during pregnancy [3]. A general decrease in sexual desire is observed during the first trimester of pregnancy; different patterns are observed in the second trimester, and, it again decreases in the third trimester [4]. Average number of times couples have intercourse per month reaches from 8.6 before pregnancy to 6.9 times per month in the first trimester, 5.4 times in the second, and 2.5 times in the third trimester of pregnancy [5].

Some factors affect sexual relationship during pregnancy, including fear of injury to the fetus [4], common complaints of pregnancy such as nausea and vomiting, the wife’s avoidance to have sex, sexual pain [6], post sex problems such as vaginal bleeding and abdominal cramp, as well as a reduced sexual desire and sexual pleasure during pregnancy [2]. In addition, pregnancy increases sexual dysfunction in women [4], and sexual dysfunction increases with the progress of pregnancy [5].

Sexual dysfunction is defined as disorders of libido, arousal, sexual pain, and orgasm [7]. Sexual dysfunction among pregnant women in Iran was rated as 60 to 79% [8, 9]. Sexual function during pregnancy can affect the quality of marital relationships [4], and, it plays an important role in sustainability of marital life [10]. Also, the quality of the couple’s relationship with each other influence on their sexual function. Sexual desire of women depending on how much marital relationship satisfaction [11]. Sexual dysfunction might provide the grounds for marital conflicts [6] and divorce by its negative effect on marital adjustment and interpersonal communication between couples [10].

Different factors can contribute to sexual dysfunction, including: education level, living with other people in the same house, unwanted pregnancy [6], couples’ age [8, 12], length of marriage [8], sufficiency of income [12] and intimate partner violence (IPV) [6]. Sexual dysfunction is more common in women who experience recent violence by their intimate partner [13, 14].

Violence is a common problem during pregnancy, and, some women might experience it for the first time during pregnancy [1]. They may have become pregnant with their partner’s sexual coercion, threats and physical violence [15]; however, sexual health requires having a sexual relationship without coercion and threat [1].

In Iran, the age structure is young, and about a quarter of the population are in the 18–29 year old group, which is regarded as a young age group [16]. Therefore, about 60% of births to Iranian couples are in this age group [17]. Given the changes in Iran’s demographic policy on population growth in recent years, it is expected to see a rising trend in birth rates [18].

There are numerous studies on women’s sexual function during pregnancy in Iran [8, 9] and the world [3, 6]. However, there is no study in Iran that exclusively examines the sexual function of young pregnant women in the 18–29 age group. In addition, few studies are conducted on the effect of intimate partner violence on women’s sexual function [13, 14], and, this has been neglected especially during pregnancy. With regard to the effect of pregnancy on sexual function, IPV, and variable patterns of sexual function during pregnancy, as well as considering changes in Iran’s demographic policy on population growth and increase in pregnancy rate, more studies should be conducted on sexual function and violence and their relationship during pregnancy. This study intended to investigate women’s sexual function and its relation with intimate partner violence, as well as the relationship between demographic variables and sexual function during the second trimester of pregnancy.

## Methods

### Study design and participants

In this cross-sectional study, 346 primigravida who visited the health centers of Tabriz (Iran) to receive routine pregnancy care, were investigated during June to October 2014. Tabriz with about 1.7 million population is the capital city of the east-Azerbaijan province. This city has 81 health centers. These centers cover more than two-thirds of pregnant women in the city. All services are provided free of charge in these centers [19, 20]. Inclusion criteria were the age between 18 to 29, being 24 to 30 weeks pregnant, length of marriage between 1 and 5 years, living with the spouse during the past year, the ability to read and write, and the desire to participate in the study. Exclusion criteria included having a spouse who was coping with chronic diseases (diabetes, hypertension, and heart disease), current experience of depression and mental diseases, a history of infertility in the current intimate partnership, the experience of stressful events (death or car accident involvement of a family member) during the past year, current spousal addiction, and having a criminal record.

### Sample size

Using G-Power software, the total sample size was determined to be ploring the link between daily relationship quality, sexual des8 considering moderate correlation between sexual function and sexual violence (*r* = 0.208) [21] and two tailed test (95% CI, and power 80%). The design effect was considered for the sample size due to using the cluster sampling method in this survey. So regarding the minimum design effect of 1.5, the study sample size was 327 women. This study was conducted on 346 women.

### Sampling

Pregnant women entered the study using two-stage cluster sampling, after the financial and ethical approval of the study at Tabriz university of Medical Sciences. First, 10 health centers were selected randomly among 39 urban health centers, and 11 centers were selected among 42 rural health centers. Then, a quota was assigned to each center according to the sample size, and the number of women covered by each center. A list of qualified pregnant women was extracted from health centers, and, based on the quota of each center; participants were randomly selected from the list. Since most pregnant women in Iran receive pregnancy care at health centers, and, their health records are kept there, their phone number has been recorded there too. Using these numbers, they were called, and, while explaining the research, the benefits of conducting it, and the confidentiality of information, these women were invited to visit the health centers at a specified time. Then, they were checked for inclusion and exclusion criteria, and the eligible participants were asked to sign the informed consent, and to fill the self-report questionnaires in a private room. We have referred the participants with IPV or sexual dysfunction to health care providers for appropriate actions.

### Data collection tools

The data collection tools consisted of a socio-demographic questionnaire and, the Female Sexual Function Index (FSFI) and Revised conflict tactics scales (CTS2).

The socio-demographic questionnaire consisted of: woman and husband age, marriage duration, woman and husband educational level, woman employment status and sufficiency of income.

### Female sexual function index (FSFI)

Female Sexual Function Index (FSFI) was designed to investigate women’s sexual function during past month, which is based on physiological phases of sexual function. The questionnaire contains 19 questions organized in 6 subdomains, namely sexual desire, arousal, lubrication, orgasm, satisfaction, and pain. The answers are scored from 0 to 5. Scores of each subdomain are obtained by the sum of the scores of questions in each subdomain and multiplying it with the factor. The total score is obtained from the sum of the scores of 6 subdomains. The higher score indicates the better sexual function. The maximum score for each subdomain is 6, which makes the highest score 36 [22]. The cut-off point for the total scale was 28, and, it was 3.3, 4.3, 4.3, 4.3, 8.3, and 8.3 for sexual desire, arousal, lubrication, orgasm, satisfaction, and sexual pain respectively. Scores over the cut-off point indicate better sexual function. Reliability of this questionnaire has been approved in Iran [23], and it has been used in various Iranian studies [9, 24].

### Revised conflict tactics scales (CTS2)

Revised conflict tactics scales (CTS2) was designed to investigate IPV during the past year. However, it could be applied for any time, and, it was used to investigate violence within the past 6 months. It consists of 78 questions and 39 items. Each question is a pair, where odd questions ask violence of women against men, and even questions ask violence of men against women. It has 5 subdomains, negotiation, psychological aggression, physical assault, sexual coercion and injuries. In five subdomains of the questionnaire, except negotiation subdomain, chronicity of violence is measured as being mild or severe. There are eight options (0 to 7) for each question that measures the frequency of violence (to score it, a median score is considered for each option. Zero, 1 and 2 have a median point similar to the number itself.) namely, 0 = never, 1 = once, 2 = twice, 3 = 3–5 times (the median point is 4), 4 = 6–10 times (the median point is 8), 5 = 11–20 times (the median point is 15), 6 = more than 20 times (the median point is 25), 7 = did not happen in the past year, but happened prior to that (the median point is 25). To measure the frequency of various dimensions of violence, if a person has selected 1 to 6 scores from the questions of a special subdomain, it means violence has occurred. If they select zero or 7 for all subdomains, it means the lack of violence for that subdomain. To measure the total violence, all subdomains of violence except the negotiation subdomain were summed up and the total violence was calculated [25]. The reliability of this instrument in Iran was evaluated using test-retest [26].

To determine the validity of the sociodemographic characteristics questionnaire, the content validity index was used. The questionnaire was reviewed by 10 members of the faculty of Tabriz University of Medical Sciences, and, the necessary amendments were made considering their comments. Reliability of CTS2 was determined in terms of repeatability (ICC = Intra Class Correlation) and internal consistency (Cronbach’s alpha). First, twenty subjects from the target group filled the questionnaire, and, they were invited to complete the questionnaire for the second time after 10 days. Repeatability (ICC) was between 93 and 99%, and, Cronbach’s alpha (consistency) was between 70 and 87%.

### Data analysis

The data were analyzed using SPSS 21. To explain sociodemographic data and sexual function and IPV descriptive statistics, including frequency and percentage, mean and standard deviation were employed. To determine the predictive variables for sexual dysfunction, adjusted binary logistic regression were used. First, the relationship between age of couples, the sufficiency of income, education of the couples, length of marriage, women’s employment, gestational age, receiving pre-pregnancy care, unwanted pregnancy, overall IPV and its subdomains with sexual dysfunction were assessed by chi-square test. Then, then, the variables of sufficiency of income, education of the couples, length of marriage, unwanted pregnancy and overall IPV that were correlated with sexual function with *p* <  0.2 based on chi-square test entered into the multivariate binary logistic regression. The significance level in the tests was considered to be less than 0.05.

## Results

Mean age of the participants was 23.5 year and their husbands’ age were 28.4 year, also duration of marriage was 2.3. About half of the women (45.1%) had a diploma and half of the husbands (46.2%) had a less than diploma. The majority of young women (93.4%) were housewife. About three - fourths of young women (73.1%) had some extent sufficiency income. The mean gestational age was 26.6 weeks. The frequency of unwanted pregnancy was 10% and only about 10% participants had received pre-pregnancy care (Table 1).

**Table 1:** Socio-demographic characteristic in young pregnant women (*n* = 346)

Characteristics	N (%)^a^
**Age** (Years)	
18–21	111 (32.1)
22–25	128 (37)
26–29	107 (30.9)
Mean (SD)^b^	23.5 (3.5)
**Educational level**	
Less than diploma	102 (29.5)
Diploma (12 years)	156 (45.1)
University	88 (25.4)
**Marriage duration** (Years)	
1	119 (34.4)
2	87 (25.1)
3–5	140 (50.5)
Mean (SD)^b^	2.3 (1.3)
**Gestational age** Mean (SD)^b^	26.6 (2.1)
**Unwanted pregnancy**	35 (10.1)
**Husband age** (Years)	
18–25	79 (22.8)
26–29	134 (38.7)
≥ 30	133 (38.4)
Mean (SD)^b^	28.4 (3.8)
**Husband Educational level**	
Less than diploma	160 (46.2)
Diploma (12 years)	115 (33.2)
University	71 (20.5)
**Sufficiency income**	
Completely	38 (11.0)
To some extent	253 (73.1)
Absolutely not	55 (15.9)
**Occupation,** Housewife	232 (93.4)
**Receiving pre-pregnancy care**	34 (9.8)

^a^Number (percent)

^b^Standard deviation

The mean (SD = standard deviation) of total sexual function score was 25.4 (5.9) from the attainable score of 2–36. The lowest and the highest mean (SD) scores in sub-domains of sexual function were in sexual desire 3.5 (1.1) and sexual satisfaction 5.1 (1.0), respectively. Prevalence of total sexual dysfunction was 66%. The most common prevalence of sexual dysfunction was in the sub domain of desire (38.4%), followed by pain (37%) and arousal (29.8%) respectively and the lowest prevalence of sexual dysfunction was in the sub-domain of sexual satisfaction (12.1%) (Table 2).

**Table 2:** Sexual function and frequency of sexual dysfunction in young pregnant women (*n* = 346)

Sexual function	Mean (SD)^a^	Sexual dysfunction	N (%) ^b^
**Desire**	3.5 (1.1)	**Desire**	133 (38.4)
**Arousal**	3.9 (1.3)	**Arousal**	103 (29.8)
**Lubrication**	4.4 (1.3)	**Lubrication**	55 (15.9)
**Orgasm**	4.4 (1.4)	**Orgasm**	62 (17.9)
**Satisfaction**	5.0 (1.0)	**Satisfaction**	42 (12.1)
**Pain**	4.1 (1.7)	**Pain**	128 (37.0)
**Total sexual function score**	25.4 (5.9)	**Total sexual dysfunction**	228 (65.9)

Possible range scores for all domains are 0–6 except for the desire which it is 1.2–6 and for the total function is 2–36, higher scores show better sexual function. The cut-off points for sub domain and sexual dysfunction were as follows: sexual desire ≤3.3, sexual arousal ≤3.4, lubrication ≤3.4, sexual pain ≤3.8, orgasm ≤3.4, sexual satisfaction ≤3.8 and sexual dysfunction ≤28

^a^Standard Deviation

^b^Number (percent)

The prevalence of overall IPV against young women was 63%. The most common type of IPV experienced by women was psychological aggression (56.6%), followed by physical assault (25.7%), sexual coercion (22.3%), and injury (8.4%). Based on the results of chi-square test, there was a significant statistical relationship between sexual dysfunction, overall IPV, psychological aggression, physical assault (*p* <  0.001), sexual coercion (*p* = 0.024) and injury (*p* = 0.016**)** (Table 3).

**Table 3:** Prevalence of intimate partner violence in young pregnant women and its correlation with sexual dysfunction (*n* = 346)

Intimate partner violence	N (%)^**a**^	The relationship With sexual dysfunction N (%)^**a**^; ***p***-value ^b^	
Psychological aggression			< 0.001
Yes	196 (56.6)	147 (75.0)	
No	150 (43.4)	81 (54.0)	
**Physical assault**			**< 0.001**
Yes	89 (25.7)	75 (84.3)	
No	257 (74.3)	153 (59.5)	
**Sexual coercion**			**0.024**
Yes	77 (22.3)	59 (76.6)	
No	269 (77.7)	169 (62.8)	
**Injury**			**0.016**
Yes	29 (8.4)	25 (86.2)	
No	317 (91.6)	203 (64.0)	
**Overall**			**< 0.001**
Yes	218 (63.0)	163 (74.8)	
No	128 (37.0)	65 (50.8)	

^a^number (percent)

^b^Chi-square test

Based on the results of chi-square test, there was a significant statistical relationship between sexual dysfunction and length of marriage (*p* <  0.05), In addition, based on the results of chi-square for trend test, there was a significant statistical relationship between sexual dysfunction, adequacy of income and the husband’s education level (*p* <  0.05) (Table 4).

**Table 4:** Correlation between sexual dysfunction and socio-demographic characteristic and intimate partner violence (*n* = 346)

Characteristics	Sexual dysfunction	
	N (%)^a^	*p*-value
**Age** (Years)		0.928^b^
18–21	72 (64.9)	
22–25	84 (65.6)	
26–29	72 (67.3)	
**Educational level**		0.135^c^
Less than diploma	72 (70.6)	
Diploma (12 years)	103 (66.0)	
University	53 (60.2)	
**Husband Educational level**		**0.025**^**c**^
Less than diploma	119 (74.4)	
Diploma (12 years)	64 (55.7)	
University	45 (63.4)	
Completely	44 (80.0)	
**Unwanted pregnancy**		0.139^b^
No	201 (64.4)	
Yes	27 (77.1)	
**Receiving pre-pregnancy care**		0.593^b^
No	21 (61.8)	
Yes	207 (66.3)	
**Husband age** (Years)		0.742^b^
18–25	53 (67.1)	
26–29	85 (63.4)	
≥ 30	90 (67.7)	
**Employment**		0.943^b^
Housewife	213 (65.9)	
Employee	15 (65.2)	
**Sufficiency of income**		**0.001**^c^
Absolutely not	17 (44.7)	
To some extent	167 (66.0)	
Completely	44 (80.0)	
**Marriage duration** (Years)		**0.028**^b^
1	69 (58.0)	
2	66 (75.9)	
3–5	93 (66.4)	
**Gestational age** (week)		0.509^b^
24–26	119 (64.3)	
27–30	109 (67.7)	

^a^number (percent)

^b^Chi-square test

^c^Chi-square for trend test

Based on the results of logistic regression, sexual dysfunction among women without IPV was less than those experiencing IPV (Odds Ratio (OR) = 0.4, 95% Confidence Interval (CI) = 0.2 to 0.6, *p* <  0.001), and, sexual dysfunction among women with adequate income was less than those with inadequate income (OR = 0.2, 95% CI = 0.1 to 0.6, *p* = 0.005). In addition, sexual dysfunction among women whose husband has a diploma was half compared to those with less than a high school diploma (OR = 0.5, 95% CI = 0.3 to 0.9, *p* = 0.028), and, sexual dysfunction among women who were married for over 2 years, was twice those who were married for a year (OR = 1.9, 95% CI = 1.0 to 3.7, *p* = 0.044) (Table 5).

**Table 5:** Correlation between sexual dysfunction and intimate partner violence and socio-demographic characteristic

Variable	OR (95% CI)^**a**^	***p***-value
**Intimate partner violence**		
Yes **(Ref)**	1	
No	0.4 (0.2 to 0.6)	**< 0.001**
**Sufficiency of income for expenses**		
Absolutely not **(Ref)**	1	
Completely	0.2 (0.1 to 0.6)	**0.005**
to some extent	0.5 (0.3 to 1.2)	0.129
**Educational level**		
Less than diploma **(Ref)**	1	
Diploma (12 years)	1.0 (0.5 to 1.8)	0.895
University	0.7 (0.3 to 1.4)	0.333
**Husband Educational level**		
Less than diploma **(Ref)**	1	
Diploma (12 years)	0.5 (0.3 to 0.9)	**0.028**
University	0.9 (0.4 to 1.8)	0.744
**Marriage duration (years)**		
1 **(Ref)**	1	
2	1.9 (1.0 to 3.7)	**0.044**
3–5	1.6 (0.9 to 2.8)	0.072
**Unwanted pregnancy**		
Yes **(Ref)**	1	
No	1.7 (0.7 to 4.2)	0.249

-The results are based on Multivariate logistic regression analysis with backward strategy

^a^Odds Ratio (95% Confidence Interval)

## Discussion

To our knowledge, this study was the first research conducted in Iran that investigated the association between sexual function, intimate partner violence, and socio-demographic characteristics of pregnant women between the ages of 18–29 years.

In the present study, 66% of young pregnant women experienced sexual dysfunction. The highest rate of sexual dysfunction was observed in sexual desire, arousal, and sexual pain respectively, and the lowest rate of sexual dysfunction was related to sexual satisfaction. Prevalence of sexual dysfunction in the second trimester of pregnancy has been 23 to 51% in various studies conducted in Iran and the world [3, 9, 27]. The prevalence of sexual dysfunction in the third trimester of pregnancy was 30% in women aged between 18 and 40 as reported by Jamali et al. [9], 40% in women aged between 19 and 31 as reported by Ahmed et al. [3], and over 34% in women aged 20 as reported by Leite et al. [28]. Bostani et al. [29] reported prevalence of sexual dysfunction in the third trimester of pregnancy among women aged between 15 and 45 as 26%, where the highest prevalence of sexual dysfunction was observed in the sexual desire subdomain (24%), and then, satisfaction from orgasm (23%), and orgasm (15%), and, the lowest prevalence of sexual dysfunction was reported in the lubrication subscale (10%). In the above-mentioned studies, the prevalence of sexual dysfunction was lower compared to the present study. In addition, in the study by Bostani et al., the highest prevalence of sexual dysfunction was related to sexual desire that complied with the results of the present study, and, the lowest prevalence of sexual dysfunction was related to lubrication that did not comply with the results of the present study. Sexual dysfunction has an extensive spectrum, including sexual desire disorder, sexual arousal disorder, orgasm disorder, dyspareunia and vaginismus [30]. In addition, using different research methods, questionnaires, inclusion and exclusion criteria, demographic variables as well as cultural and religious differences may lead to different prevalence of sexual dysfunction in the present study compared to other studies.

In the present study, prevalence of overall IPV was 63%. Relatively similar results have been reported from other cities of Iran [31, 32], which can be attributed to the use of similar instrument (CTS2) or similar setting. Envuladu et al. has been reported IPV 31.8% in Nigeria by semi structured interviewer administered questionnaire [15]. This observed difference between the result of the present study and Envuladu et al.’s study maybe due to the different instruments used to assess IPV. The results of this study showed that psychological aggression had the highest prevalence in IPV against young women. This finding is consistent with other studies in Iran [31–33]. Also, according to obtained results by Martin et al. in North Carolina, pregnancy was not associated with significant increases in the rates of physical assault or injuries, but psychological aggression was increased [34]. Envuladu et al. in Nigeria showed that the most prevalent form of violence was being forced to have sexual intercourse and threat of harming and physical slapping were less common [15]. The cultural and religious differences may lead to different result in the present study compared Nigeria study.

The mean score of sexual function in the present study was 25.4 where the lowest and the highest score were related to sexual desire and sexual satisfaction, respectively. Consistent with our results, Aslan et al. [5] and Leite et al. [28] reported similar results in pregnant women except the highest score in the Leite et al.’s study was related to vagina wetness [28]. Sexual desire may decrease during pregnancy due to the fear of abortion, rupture of the amniotic sac and damage to the embryo or fetus. On the other hand, vascular congestion of the female reproductive organs occurs in the second trimester of pregnancy, and, women may experience a more fulfilling for the first time [4], and, these factors may increase their satisfaction from sexual relationship.

The results of this study showed that despite women experiencing sexual dysfunction in subdomains of arousal, lubrication, orgasm, pain and arousal, women’s levels of sexual satisfaction have not suffered nearly as much. The culture and beliefs may influence on sexual satisfaction [35]. Iranian women define sexual satisfaction as having no problem with their sex life, being loved by their husband, the husband’s sexual contentment [36] and they feel satisfied with sex through meeting sexual needs of husband [37, 38]. Also, family is a sacred foundation in Islam, and in Iran society, marriage is holy and women consider sex as a means for stability of their marriage, therefore many of women despite having no pleasure in sex seems to feel satisfied with sex, since the person is satisfied with the whole system and intends to keep marriage [37].

In this study, women without IPV showed less sexual dysfunction and there was a significant statistical relationship between sexual dysfunction, psychological aggression, physical assault, sexual coercion and injury. In the study conducted by Hastuti et al. on women of reproductive age in Indonesia, it was indicated that women who experience intimate partner violence were four times more likely to show sexual dysfunction [13]. In addition, Ipekten Alaman and Yildiz in Turkey reported that women who experience IPV show more sexual dysfunction, and suffer specially from orgasm disorder [39]. In Iran, Jamali et al. showed that increased IPV led to sexual dysfunction [14]. Results of their study comply with the results of the present study. Ipekten Alaman and Yildiz reported that when women were passive in terms of sexuality or when their husbands were not sexually satisfied, their husbands violence behaviors increased, and women expressed that they occasionally had sexual intercourse unwillingly since they were afraid of their spouse’s violent reactions [39]. In general, the inability to respond sexual needs plays an important role in the couple’s dissatisfaction. Not meeting each other’s sexual needs that is likely due to sexual dysfunction, may damage the couple’s relationship.

In the present study, women with adequate income showed less sexual dysfunction compared to women with inadequate income. In the study conducted by Nikoobakht et al. in Qazvin, Iran, a significant statistical relationship was observed between better sexual function and higher income in 18-year-old women and older [12]. Higher income can lead to greater satisfaction from life, sexual satisfaction, and increased quality of sexual life, and, it can reduce sexual dysfunction. In the study conducted by Smith et al., higher level of education was predictive factor for a lower sexual function in women at menopause age, which does not comply with the results of the present study [40, 41]. This is likely due to the age difference of the participants included in each of these studies.

In this study, sexual dysfunction was lower among women whose husbands had diploma compared to those with less than a high school diploma. In the study conducted by Jafarzadeh-Esfehan et al., there was a significant statistical relationship between sexual dysfunction and the spouse’s education, and, women whose husbands had diploma or less than a high school diploma, showed higher sexual dysfunction [24]. In the study conducted by Kucukdurmaz et al., sexual dysfunction decreased by increased level of education [42]. The couple’s awareness of sexual dysfunction may likely increase as their level of education rises, and, they may seek counseling and therapy that reduce sexual dysfunction.

The present study indicated that sexual dysfunction among women who were married for 2 years was higher than those who were married for 1 year. Aboozari et al. [8] and Jafarzadeh-Esfehan et al. [24] showed that sexual dysfunction reduces by the length of marriage. However, their results do not comply with the results of the present study. Love at the beginning of marriage may result in less attention paid to sexual dysfunction in couples, which may improve sexual satisfaction in them.

Based on the results of this study, it is necessary that health care providers to take measures for screening sexual dysfunction and IPV during pregnancy. Also, health care providers can train effective communication skills for couples to elimination of marital conflicts and dissatisfaction. Considering the association between sufficiency of income and education level with sexual dysfunction, the policymakers should increase family income with proper planning and provide conditions for people to achieve the highest level of education.

The first strength of the present study was to investigate sexual dysfunction among young pregnant women in Iran for the first time. Another strength of this study was that it employed a standard questionnaire, which was used extensively in Iran and the world, to investigate sexual function and violence.

The first limitation of the present study was its inability to compare sexual function before and after pregnancy due to its cross-sectional nature. In addition, due to the cross-sectional nature of the present study, it was not possible to investigate the causal relationship between intimate partner violence and demographic variables with sexual function. Another limitation of the present study was that only those women referring the health centers were investigated and those who did not refer were not investigated, and, it was not possible to generalize the results to the whole society. Therefore, it is suggested to manage these limitations in future studies.

## Conclusion

In this study, the frequency of sexual dysfunction was considerable among young pregnant women. The highest and lowest frequency of sexual dysfunction was related to sexual desire and sexual satisfaction respectively. Prevalence of IPV was high and in IPV sub-domains psychological aggression was common. In addition, there was a significant statistical relationship between sexual dysfunction and IPV, the adequacy of income, the spouse’s education, and length of marriage. Sexual function is an important factor for a sustainable marriage and satisfaction from life, and, it plays an important role in reducing marital conflicts and divorce. Therefore, it is suggested to consider the couple’s training and consulting in the pregnancy cares to improve their sexual function and decrease IPV.

## Data Availability

The datasets generated and analyzed during the current study are available in the Research Center, Tabriz University of Medical Sciences.

## Abbreviations

IPV: Intimate Partner Violence
FSFI: Female Sexual Function Index
CTS2: Conflict Tactics Scale
SD: Standard Deviation
OR: Odds Ratio
